# Crystal structure of (2*E*)-3-[4-(di­methyl­amino)­phen­yl]-1-(thio­phen-2-yl)prop-2-en-1-one

**DOI:** 10.1107/S2056989017003437

**Published:** 2017-03-07

**Authors:** Gabriela Porto de Oliveira, Leandro Bresolin, Darlene Correia Flores, Renan Lira de Farias, Adriano Bof de Oliveira

**Affiliations:** a Universidade Federal do Rio Grande (FURG), Escola de Química e Alimentos, Rio Grande, Brazil; b Universidade Estadual Paulista (UNESP), Instituto de Química, Araraquara, Brazil; c Universidade Federal de Sergipe (UFS), Departamento de Química, São Cristóvão, Brazil

**Keywords:** crystal structure, chalcone thio­phene derivative, Hirshfeld surface analysis, *in silico* evaluation

## Abstract

The equimolar reaction between 4-(di­methyl­amino)­benzaldehyde and 2-acetyl­thio­phene in basic ethano­lic solution yields the title compound, C_15_H_15_NOS, whose mol­ecular structure matches the asymmetric unit. The mol­ecule is not planar, the dihedral angle between the aromatic and the thio­phene rings being 11.4 (2)°. In the crystal, mol­ecules are linked by C—H⋯O and weak C—H⋯S inter­actions along [100], forming *R_2_^2^*(8) rings, and by weak C—H⋯O inter­actions along [010], forming chains with a *C*(6) graph-set motif. In addition, mol­ecules are connected into centrosymmetric dimers by weak C—H⋯π inter­actions, as indicated by the Hirshfeld surface analysis. The most important contributions for the crystal structure are the H⋯H (46.50%) and H⋯C (23.40%) inter­actions. The crystal packing resembles a herringbone arrangement when viewed along [100]. A mol­ecular docking calculation of the title compound with the neuraminidase enzyme was carried out. The enzyme shows (*ASN263*)N—H⋯O, (*PRO245*)C—H⋯*Cg*(thio­phene ring) and (*AGR287*)C—H⋯N inter­molecular inter­actions with the title compound. The crystal structure was refined as a two-component twin with a fractional contribution to the minor domain of 0.0181 (8).

## Abstract 2

In the title chalcone-thio­phene derivative, the dihedral angle between the aromatic and the thio­phene rings is 11.4 (2)°. In the crystal, mol­ecules are linked by C—H⋯O and C—H⋯S weak inter­actions along [100], forming rings of *R_2_^2^*(8) graph-set motif, by C—H⋯O weak inter­actions along [010], forming *C*(6) chains, and by weak H(meth­yl–group)⋯*Cg*(thio­phene ring) inter­actions into dimers; the crystal packing resembles a herringbone arrangement when viewed along [100]. A mol­ecular docking calculation of the title compound with the neuraminidase enzyme was carried out.

## Chemical context   

Chalcone derivatives are compounds with an aromatic conjugated enone as the main fragment and are synthesized by hydroxide-catalysed aldol condensation between an aromatic aldehyde and a ketone. Some of the first preparative methods of the aldol condensation were reported in the second half of the 19th Century (Claisen & Claparède, 1881 ▸; Schmidt, 1881 ▸) and the experimental procedure remains the same to the present time. Chalcone compounds can be obtained from a great number of starting materials, resulting in a class of compounds with a wide range of properties and applications, specially in the medicinal chemistry. Several 4-di­alkyl­amino­chalcones have shown anti­proliferative activity on cancer cell lines and one method to monitor the chalcone–protein inter­action, *e.g.* tubulin proteins, is the chalcone’s fluorescence (Zhou *et al.*, 2016 ▸). Another example of the pharmacological background for the title compound and its derivatives is the anti-influenza viral activity through the neuraminidase enzymatic inhibition *in vitro* (Kinger *et al.*, 2012 ▸). Thus, the crystal structure determination of chalcone-based mol­ecules is an intensive research area, in particular for its contributions in medicinal chemistry. As part of our studies in this field, we describe herein the crystal structure, the Hirshfeld surface analysis and the mol­ecular docking evaluation of the title compound.
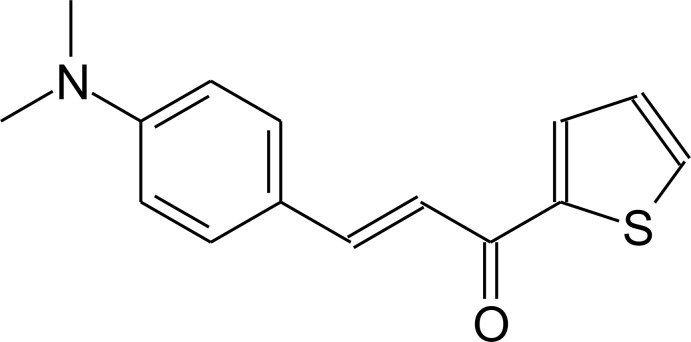


## Structural commentary   

**Figure 1:**
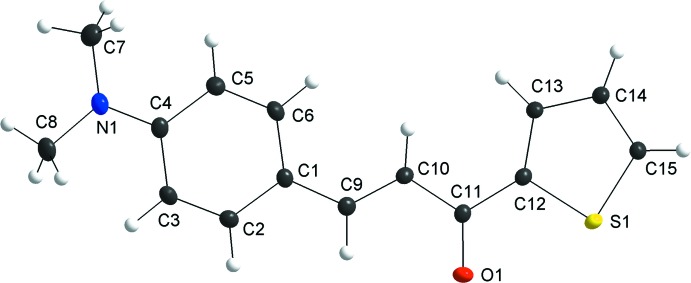
The mol­ecular structure of the title compound, showing displacement ellipsoids drawn at the 40% probability level.

In the crystal structure of the title compound, a chalcone-thio­phene derivative, the asymmetric unit contains one crystallographically independent mol­ecule (Fig. 1 ▸). The mol­ecule is not planar: the r.m.s deviations from the mean plane of the non–H atoms range from −0.158 (3) Å for C3 to 0.1318 (15) Å for S1 and the dihedral angle between the benzene and thio­phene rings amounts to 11.4 (2)°. In addition, the plane through the amino group atoms (C7/C8/N1) is rotated by 9.7 (6)° with respect to the plane of the aromatic ring. Finally, the mol­ecule shows the *E* configuration about the C9—C10 bond.

## Supra­molecular features   

**Figure 2:**
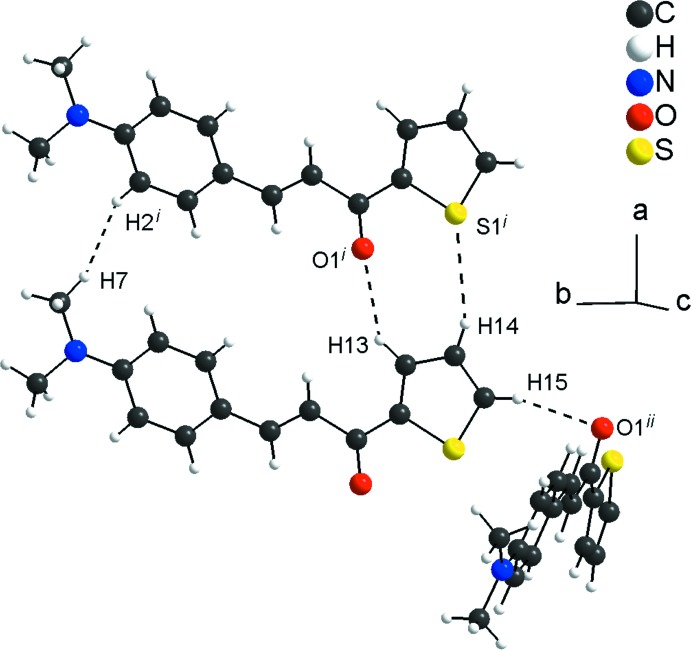
Graphical representation of the weak inter­molecular C—H⋯O, C—H⋯S and H⋯H inter­actions (dashed lines) in the crystal structure of the title compound. [Symmetry codes: (i) *x* + 1, *y*, *z*; (ii) −*x* − 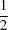, *y* − 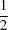, −*z* + 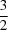.]

**Figure 3:**
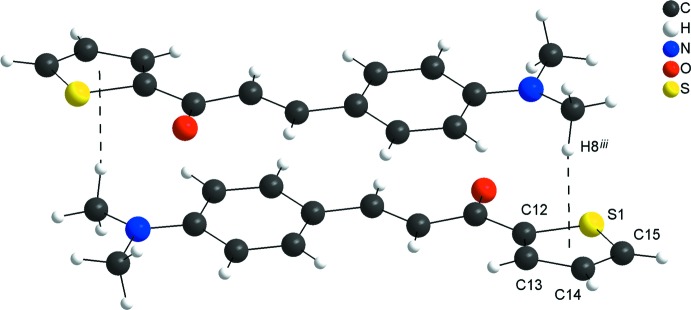
Graphical representation of the weak inter­molecular C—H⋯π inter­actions (dashed lines) in the crystal structure of the title compound, forming a centrosymmetric dimer. [Symmetry code: (iii) −*x*, −*y*, −*z* + 1.]

In the crystal, the mol­ecules are connected by very weak C13—H13⋯O1^i^ and C14—H14⋯S1^i^ hydrogen-bonding inter­actions (see Table 1 ▸ for symmetry codes), forming rings with an *R_2_^2^*(8) graph-set motif. The *R_2_^2^*(8) rings are the subunits of the periodic arrangement along [100] and one very weak H7⋯H2^i^ contact is also observed [H⋯H = 2.26 Å]. The mol­ecular units are also linked by very weak C15—H15⋯O1^ii^ links into chains along [010] with a *C*(6) graph-set motif (Fig. 2 ▸; Table 1 ▸). Additionally, the mol­ecules are connected into centrosymmetric dimers by very weak C—H⋯π inter­actions involving the thio­phene ring (Fig. 3 ▸; Table 1 ▸). The inter­molecular contacts are slightly longer than the sum of the van der Waals radii for the respective atoms (Bondi, 1964 ▸; Rowland & Taylor, 1996 ▸) and suggest weak inter­actions only.

## Hirshfeld surface analysis   

**Figure 4:**
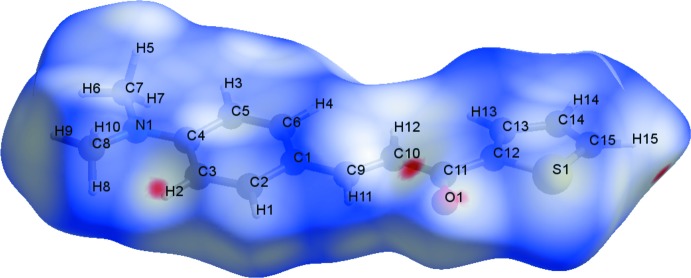
A graphical representation of the Hirshfeld surface (*d*_norm_) for the title compound. The surface is drawn with transparency and all atoms are labelled. The surface regions with strongest inter­molecular inter­actions for atoms H2, H15 and O1 are shown in magenta.

The Hirshfeld surface analysis (Hirshfeld, 1977 ▸) of the crystal structure suggests that the contribution of the H⋯H inter­molecular inter­actions to the crystal packing amounts to 46.50% and the contribution of the H⋯C inter­actions amounts to 23.40%. Other important inter­molecular contacts for the cohesion of the structure are (values given in %): H⋯O = 10.80 and H⋯S = 10.00. Graphical representations of the Hirshfeld surface with transparency and labelled atoms (Figs. 4 ▸ and 5 ▸) indicate, in a magenta colour, the locations of the strongest inter­molecular contacts, *e.g.* the H2, H7, H13, H15 and O1 atoms. The C—H⋯π inter­action is also well represented in the Hirshfeld surface (for details, compare Figs. 3 ▸ and 5 ▸). The H⋯H, H⋯C, H⋯O and H⋯S contributions to the crystal packing are shown as a Hirshfeld surface two-dimensional fingerprint plot with cyan dots. The *d*_e_ (*y* axis) and *d*_i_ (*x* axis) values are the closest external and inter­nal distances (values given in Å) from given points on the Hirshfeld surface contacts (Fig. 6 ▸; Wolff *et al.*, 2012 ▸).

## Mol­ecular docking evaluation   

**Figure 5:**
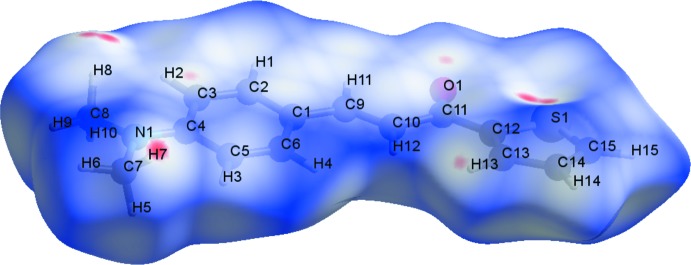
A graphical representation of the Hirshfeld surface (*d*_norm_) for the title compound. The surface is drawn with transparency and all atoms are labelled. The surface regions with strongest inter­molecular inter­actions for atoms H7, H8, H13 and O1, and for the thio­phene ring, are shown in magenta.

**Figure 6:**
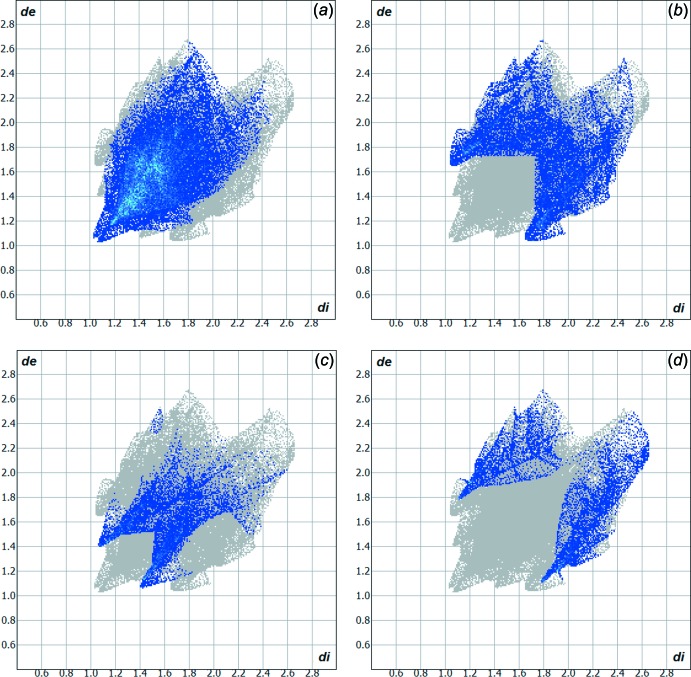
Hirshfeld surface two-dimensional fingerprint plots for the title compound showing the (*a*) H⋯H, (*b*) H⋯C, (*c*) O⋯H and (*d*) H⋯S contacts in detail (cyan dots). The contributions of the inter­actions to the crystal packing amount to 46.50, 23.40, 10.80 and 10.00%, respectively. The *d*_e_ (*y* axis) and *d*_i_ (*x* axis) values are the closest external and inter­nal distances (values in Å) from given points on the Hirshfeld surface contacts.

**Figure 7:**
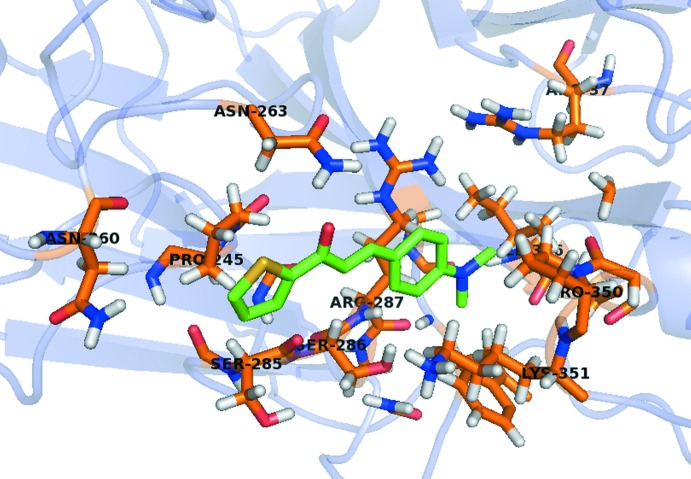
Graphical representation of the lock-and-key model for the title compound, with the mol­ecular main fragment in green, and the neuraminidase structure, with selected amino acids residues, in stick model. The structure of the enzyme is simplified for clarity.

**Figure 8:**
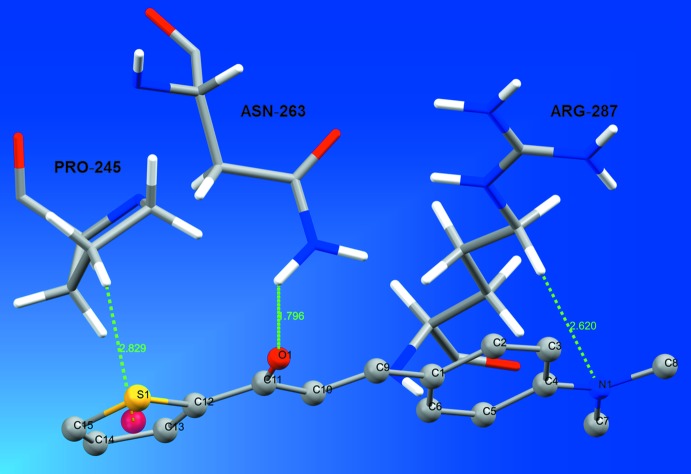
Inter­molecular inter­actions between the title compound and the neuraminidase enzyme. The inter­actions are shown as dashed lines and the structure of the enzyme is simplified for clarity.

In addition, a lock-and-key supra­molecular analysis between the neuraminidase enzyme, whose inhibition is believed to be a key point to block the influenza viral infection (Kinger *et al.*, 2012 ▸), and the title compound was performed. The semi-empirical equilibrium energy of the title compound was obtained using the PM6 Hamiltonian and the experimental bond lengths were conserved. The calculated parameters were: heat of formation = 139.28 kJ mol^−1^, gradient normal = 0.62031, HOMO = −8.96 eV, LUMO = −0.866 eV and energy gap = 7.421 eV (Stewart, 2013 ▸). The rigid mol­ecular docking was carried out with the *GOLD* software (Jones *et al.*, 1997 ▸) using the *ChemPLP* score function (Chen, 2015 ▸). The chalcone thio­phene derivative and the active site of the neuraminidase match (Fig. 7 ▸) and the structure–activity relationship can be assumed by the following observed inter­molecular inter­actions (H⋯A distance values given in Å): (*ASN263*)N—H⋯O1 (*d* = 1.796), (*PRO245*)C—H⋯*Cg*(thio­phene ring) (*d* = 2.829) and (*AGR287*)C—H⋯N1 (*d* = 2.620) (Fig. 8 ▸). More details about the *in silico* evaluation, with additional references, can be found in the *Supporting Information*. For the inter­molecular inter­actions, it is important to report that the H⋯*Cg*(thio­phene ring) contact is observed in the structure inter­pretation, by the centrosymmetric dimeric arrangement of the mol­ecules (Figs. 3 ▸ and 9 ▸), in the Hirshfeld surface analysis (Fig. 5 ▸) and in the mol­ecular docking evaluation (Fig. 8 ▸).

## Database survey   

**Figure 9:**
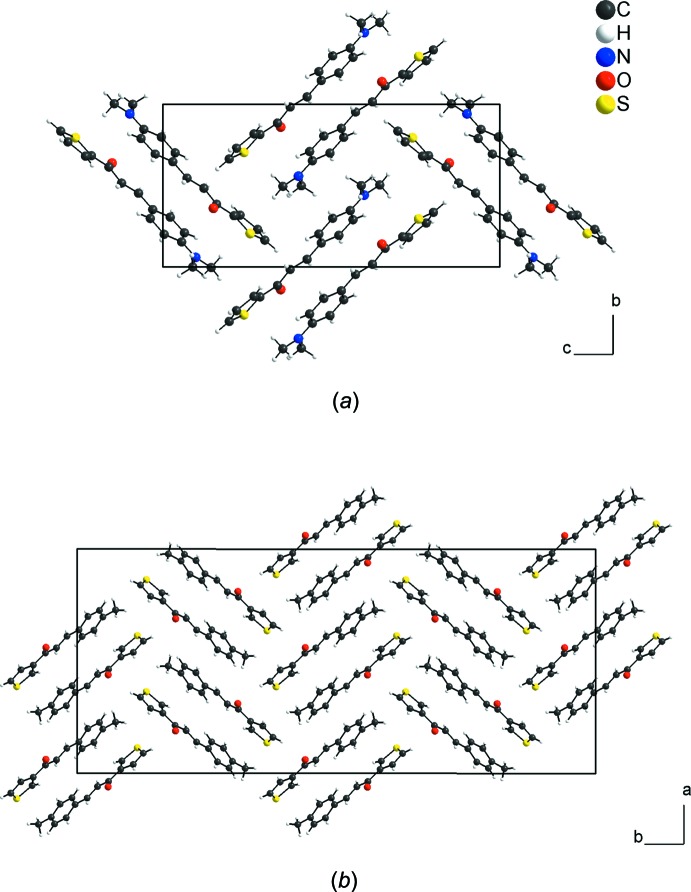
Section of the crystal structures of (*a*) the title compound viewed along [100], and (*b*) the 3-(4-methyl­phen­yl)-1-(3-thien­yl)-2-propen-1-one compound (Li & Su, 1993 ▸) viewed along [001], showing the herringbone motif.

Chalcone-thio­phene derivatives have some mol­ecular structural features in common, namely the nearly planar geometry, as a result of the *sp* ^2^-hybridized C atoms of the main fragment, and the weak inter­molecular inter­actions, *e.g.* H⋯H, H⋯C or π–π contacts. One example for comparison with the title compound is the crystal structure of the compound 3-(4-methyl­phen­yl)-1-(3-thien­yl)-2-propen-1-one (Li & Su, 1993 ▸). In both of the structures, the mol­ecules are linked by weak inter­actions into centrosymmetric dimers and the crystal packing shows a herringbone motif: for the title compound this mol­ecular arrangement is clear when looking along the [100] direction (Fig. 9 ▸ *a*) and for the above-mentioned 3-thienyl derivative, along [001] (Fig. 9 ▸ *b*).

## Synthesis and crystallization   

All starting materials are commercially available and were used without further purification. The synthesis of the title compound was adapted from a previously reported procedure (Claisen & Claparède, 1881 ▸; Schmidt, 1881 ▸; Zhou *et al.*, 2016 ▸). In a hydroxide-catalysed reaction, a mixture of 4-(di­methyl­amino)­benzaldehyde (10 mmol) and 2-acetyl­thio­phene (10 mmol) in ethanol (80 mL) was stirred under room temperature for 4 h. After cooling in an ice bath and filtering, an orange solid was obtained. Orange crystals were grown from the solution after 24 h.

## Refinement   

Crystal data, data collection and structure refinement details are summarized in Table 2 ▸. H atoms were located in a difference-Fourier map but were positioned with idealized geometry and were refined with isotropic displacement parameters using a riding model (HFIX command) with *U*_iso_(H) = 1.2*U*_eq_(C) or 1.5*U*_eq_(C) for methyl H atoms. A rotating model was used for the methyl groups. The crystal was refined as a two-component twin {twin law: two-axis (001) [105], BASF = 0.0181 (8)}.

## Supplementary Material

Crystal structure: contains datablock(s) I. DOI: 10.1107/S2056989017003437/rz5205sup1.cif

Structure factors: contains datablock(s) I. DOI: 10.1107/S2056989017003437/rz5205Isup2.hkl

SUPPORTING INFORMATION FOR THE EVALUATION OF THE CRYSTAL STRUCTURE OF (2E)-3-[4-(DIMETHYLAMINO)PHENYL]-1-(THIOPHEN-2-YL)PROP-2-EN-1-ONE AND THE NEURAMINIDASE ENZYME. DOI: 10.1107/S2056989017003437/rz5205sup3.pdf

Supporting information file. DOI: 10.1107/S2056989017003437/rz5205Isup4.cml

CCDC reference: 1535563

Additional supporting information:  crystallographic information; 3D view; checkCIF report

## Figures and Tables

**Table 1:** Hydrogen-bond geometry (Å, °) *Cg* is the centroid of the S1/C12–C15 thio­phene ring.

*D*—H⋯*A*	*D*—H	H⋯*A*	*D*⋯*A*	*D*—H⋯*A*
C13—H13⋯O1^i^	0.95	2.65	3.451 (4)	142
C14—H14⋯S1^i^	0.95	3.00	3.779 (3)	141
C15—H15⋯O1^ii^	0.95	2.57	3.291 (4)	133
C8—H8⋯*Cg* ^iii^	0.98	2.64	3.457 (4)	141

Symmetry codes: (i) 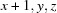; (ii) 
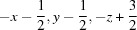
; (iii) 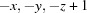.

**Table 2:** Experimental details

Crystal data	
Chemical formula	C_15_H_15_NOS
*M*_r_	257.34
Crystal system, space group	Monoclinic, *P*2_1_/*n*
Temperature (K)	120
*a*, *b*, *c* (Å)	6.2405 (4), 9.9975 (6), 20.7815 (13)
β (°)	93.097 (2)
*V* (Å^3^)	1294.65 (14)
*Z*	4
Radiation type	Mo *K*α
μ (mm^−1^)	0.24
Crystal size (mm)	0.53 × 0.16 × 0.09

Data collection	
Diffractometer	Bruker APEXII CCD area detector
Absorption correction	Multi-scan (*SADABS*; Bruker, 2014 ▸)
*T*_min_, *T*_max_	0.885, 0.979
No. of measured, independent and observed [*I* > 2σ(*I*)] reflections	50820, 3381, 3116
*R*_int_	0.031
(sin θ/λ)_max_ (Å^−1^)	0.677

Refinement	
*R*[*F* ^2^ > 2σ(*F* ^2^)], *wR*(*F* ^2^), *S*	0.068, 0.182, 1.14
No. of reflections	3381
No. of parameters	166
H-atom treatment	H-atom parameters constrained
Δρ_max_, Δρ_min_ (e Å^−3^)	1.16, −0.81

Computer programs: *APEX2* and *SAINT* (Bruker, 2014 ▸), *SHELXS97* (Sheldrick, 2008 ▸), *SHELXL2016* (Sheldrick, 2015 ▸), *WinGX* (Farrugia, 2012 ▸), *DIAMOND* (Brandenburg, 2006 ▸), *GOLD* (Chen *et al.*, 2015 ▸), *Crystal Explorer* (Wolff *et al.*, 2012 ▸), *publCIF* (Westrip, 2010 ▸) and *enCIFer* (Allen *et al.*, 2004 ▸).

## supplementary crystallographic information

### Crystal data

**Table:**

C_15_H_15_NOS	*F*(000) = 544
*M**_r_* = 257.34	*D*_x_ = 1.320 Mg m^−^^3^
Monoclinic, *P*2_1_/*n*	Mo *K*α radiation, λ = 0.71073 Å
*a* = 6.2405 (4) Å	Cell parameters from 9620 reflections
*b* = 9.9975 (6) Å	θ = 2.3–28.7°
*c* = 20.7815 (13) Å	µ = 0.24 mm^−^^1^
β = 93.097 (2)°	*T* = 120 K
*V* = 1294.65 (14) Å^3^	Block, orange
*Z* = 4	0.53 × 0.16 × 0.09 mm

### Data collection

**Table:**

Bruker APEXII CCD area detector diffractometer	3116 reflections with *I* > 2σ(*I*)
Radiation source: fine-focus sealed tube, Bruker APEXII CCD area detector	*R*_int_ = 0.031
φ and ω scans	θ_max_ = 28.8°, θ_min_ = 2.3°
Absorption correction: multi-scan (SADABS; Bruker, 2014)	*h* = −7→8
*T*_min_ = 0.885, *T*_max_ = 0.979	*k* = −13→13
50820 measured reflections	*l* = −28→28
3381 independent reflections	

### Refinement

**Table:**

Refinement on *F*^2^	Primary atom site location: structure-invariant direct methods
Least-squares matrix: full	Secondary atom site location: difference Fourier map
*R*[*F*^2^ > 2σ(*F*^2^)] = 0.068	Hydrogen site location: inferred from neighbouring sites
*wR*(*F*^2^) = 0.182	H-atom parameters constrained
*S* = 1.14	*w* = 1/[σ^2^(*F*_o_^2^) + 7.019*P*] where *P* = (*F*_o_^2^ + 2*F*_c_^2^)/3
3381 reflections	(Δ/σ)_max_ < 0.001
166 parameters	Δρ_max_ = 1.16 e Å^−^^3^
0 restraints	Δρ_min_ = −0.81 e Å^−^^3^

### Special details

**Table:**

Geometry. All esds (except the esd in the dihedral angle between two l.s. planes) are estimated using the full covariance matrix. The cell esds are taken into account individually in the estimation of esds in distances, angles and torsion angles; correlations between esds in cell parameters are only used when they are defined by crystal symmetry. An approximate (isotropic) treatment of cell esds is used for estimating esds involving l.s. planes.
Refinement. Refined as a 2-component twin.

### Fractional atomic coordinates and isotropic or equivalent isotropic displacement parameters (Å^2^)

**Table:**

	*x*	*y*	*z*	*U*_iso_*/*U*_eq_	
C1	−0.1108 (5)	0.1473 (3)	0.52979 (15)	0.0159 (6)	
C2	−0.2422 (5)	0.1940 (4)	0.47768 (16)	0.0189 (6)	
H1	−0.382513	0.157938	0.471036	0.023*	
C3	−0.1753 (5)	0.2904 (4)	0.43572 (15)	0.0193 (6)	
H2	−0.269640	0.319201	0.401045	0.023*	
C4	0.0319 (5)	0.3467 (3)	0.44378 (15)	0.0178 (6)	
C5	0.1659 (5)	0.2992 (3)	0.49602 (16)	0.0188 (6)	
H3	0.306383	0.334799	0.502939	0.023*	
C6	0.0956 (5)	0.2024 (3)	0.53690 (15)	0.0181 (6)	
H4	0.190279	0.171734	0.571104	0.022*	
C7	0.3103 (6)	0.5007 (4)	0.4112 (2)	0.0295 (8)	
H5	0.318786	0.553158	0.451058	0.044*	
H6	0.336378	0.559200	0.374572	0.044*	
H7	0.418747	0.429736	0.413870	0.044*	
C8	−0.0527 (6)	0.5041 (4)	0.35627 (17)	0.0241 (7)	
H8	−0.117365	0.435643	0.327624	0.036*	
H9	0.021999	0.570086	0.330728	0.036*	
H10	−0.165267	0.548805	0.379384	0.036*	
C9	−0.1918 (5)	0.0467 (3)	0.57189 (15)	0.0168 (6)	
H11	−0.336808	0.020903	0.562907	0.020*	
C10	−0.0899 (5)	−0.0158 (3)	0.62222 (15)	0.0163 (6)	
H12	0.056053	0.004730	0.633416	0.020*	
C11	−0.2021 (5)	−0.1147 (3)	0.65969 (15)	0.0164 (6)	
C12	−0.0788 (5)	−0.1847 (3)	0.71167 (15)	0.0155 (6)	
C13	0.1366 (5)	−0.1816 (3)	0.72937 (15)	0.0174 (6)	
H13	0.237533	−0.126760	0.709136	0.021*	
C14	0.1911 (5)	−0.2697 (3)	0.78141 (16)	0.0194 (6)	
H14	0.333099	−0.281313	0.799380	0.023*	
C15	0.0180 (5)	−0.3357 (3)	0.80267 (15)	0.0180 (6)	
H15	0.024715	−0.397159	0.837576	0.022*	
N1	0.0994 (5)	0.4417 (3)	0.40233 (14)	0.0232 (6)	
O1	−0.3960 (4)	−0.1393 (3)	0.65025 (12)	0.0220 (5)	
S1	−0.21315 (13)	−0.29494 (9)	0.75923 (4)	0.0187 (2)	

### Atomic displacement parameters (Å^2^)

**Table:**

	*U*^11^	*U*^22^	*U*^33^	*U*^12^	*U*^13^	*U*^23^
C1	0.0188 (14)	0.0149 (14)	0.0140 (14)	0.0025 (12)	0.0012 (11)	−0.0014 (11)
C2	0.0160 (14)	0.0211 (16)	0.0195 (15)	0.0028 (12)	0.0003 (12)	−0.0010 (13)
C3	0.0187 (15)	0.0221 (16)	0.0167 (14)	0.0051 (13)	−0.0014 (12)	0.0004 (13)
C4	0.0226 (16)	0.0152 (14)	0.0159 (14)	0.0039 (12)	0.0030 (12)	−0.0011 (12)
C5	0.0200 (15)	0.0170 (15)	0.0191 (15)	−0.0006 (12)	−0.0011 (12)	−0.0016 (12)
C6	0.0211 (15)	0.0179 (15)	0.0148 (14)	0.0020 (13)	−0.0029 (11)	−0.0011 (12)
C7	0.0287 (19)	0.0276 (19)	0.032 (2)	−0.0026 (16)	0.0024 (15)	0.0082 (16)
C8	0.0300 (18)	0.0202 (16)	0.0220 (16)	0.0055 (14)	−0.0009 (14)	0.0054 (13)
C9	0.0190 (15)	0.0161 (14)	0.0156 (14)	0.0017 (12)	0.0027 (11)	−0.0033 (12)
C10	0.0172 (14)	0.0158 (14)	0.0159 (14)	−0.0027 (12)	0.0011 (11)	−0.0025 (12)
C11	0.0183 (14)	0.0159 (14)	0.0150 (14)	0.0004 (12)	0.0023 (11)	−0.0018 (11)
C12	0.0168 (14)	0.0146 (14)	0.0154 (14)	−0.0017 (11)	0.0030 (11)	−0.0020 (11)
C13	0.0165 (14)	0.0174 (15)	0.0182 (15)	−0.0029 (12)	0.0004 (11)	0.0020 (12)
C14	0.0176 (14)	0.0202 (16)	0.0203 (15)	−0.0023 (12)	0.0011 (12)	0.0047 (13)
C15	0.0184 (15)	0.0199 (15)	0.0157 (14)	−0.0009 (12)	0.0001 (11)	0.0010 (12)
N1	0.0267 (15)	0.0216 (14)	0.0211 (14)	0.0009 (12)	−0.0012 (12)	0.0062 (12)
O1	0.0145 (11)	0.0256 (13)	0.0258 (12)	−0.0025 (10)	−0.0014 (9)	0.0034 (10)
S1	0.0137 (4)	0.0218 (4)	0.0210 (4)	−0.0023 (3)	0.0030 (3)	0.0038 (3)

### Geometric parameters (Å, º)

**Table:**

C1—C6	1.401 (5)	C8—H8	0.9800
C1—C2	1.403 (4)	C8—H9	0.9800
C1—C9	1.442 (4)	C8—H10	0.9800
C2—C3	1.379 (5)	C9—C10	1.348 (5)
C2—H1	0.9500	C9—H11	0.9500
C3—C4	1.412 (5)	C10—C11	1.461 (4)
C3—H2	0.9500	C10—H12	0.9500
C4—N1	1.364 (4)	C11—O1	1.240 (4)
C4—C5	1.416 (5)	C11—C12	1.469 (4)
C5—C6	1.375 (5)	C12—C13	1.375 (4)
C5—H3	0.9500	C12—S1	1.728 (3)
C6—H4	0.9500	C13—C14	1.422 (4)
C7—N1	1.445 (5)	C13—H13	0.9500
C7—H5	0.9800	C14—C15	1.359 (4)
C7—H6	0.9800	C14—H14	0.9500
C7—H7	0.9800	C15—S1	1.709 (3)
C8—N1	1.452 (4)	C15—H15	0.9500
			
C6—C1—C2	116.5 (3)	H8—C8—H10	109.5
C6—C1—C9	124.1 (3)	H9—C8—H10	109.5
C2—C1—C9	119.4 (3)	C10—C9—C1	128.9 (3)
C3—C2—C1	122.4 (3)	C10—C9—H11	115.6
C3—C2—H1	118.8	C1—C9—H11	115.6
C1—C2—H1	118.8	C9—C10—C11	120.5 (3)
C2—C3—C4	120.7 (3)	C9—C10—H12	119.7
C2—C3—H2	119.7	C11—C10—H12	119.7
C4—C3—H2	119.7	O1—C11—C10	122.8 (3)
N1—C4—C3	121.0 (3)	O1—C11—C12	119.3 (3)
N1—C4—C5	121.9 (3)	C10—C11—C12	117.9 (3)
C3—C4—C5	117.1 (3)	C13—C12—C11	130.7 (3)
C6—C5—C4	121.0 (3)	C13—C12—S1	111.0 (2)
C6—C5—H3	119.5	C11—C12—S1	118.3 (2)
C4—C5—H3	119.5	C12—C13—C14	112.3 (3)
C5—C6—C1	122.2 (3)	C12—C13—H13	123.8
C5—C6—H4	118.9	C14—C13—H13	123.8
C1—C6—H4	118.9	C15—C14—C13	112.7 (3)
N1—C7—H5	109.5	C15—C14—H14	123.6
N1—C7—H6	109.5	C13—C14—H14	123.6
H5—C7—H6	109.5	C14—C15—S1	112.1 (3)
N1—C7—H7	109.5	C14—C15—H15	124.0
H5—C7—H7	109.5	S1—C15—H15	124.0
H6—C7—H7	109.5	C4—N1—C7	120.9 (3)
N1—C8—H8	109.5	C4—N1—C8	120.1 (3)
N1—C8—H9	109.5	C7—N1—C8	117.8 (3)
H8—C8—H9	109.5	C15—S1—C12	91.87 (16)
N1—C8—H10	109.5		
			
C6—C1—C2—C3	−0.9 (5)	O1—C11—C12—C13	175.7 (3)
C9—C1—C2—C3	179.7 (3)	C10—C11—C12—C13	−5.9 (5)
C1—C2—C3—C4	0.0 (5)	O1—C11—C12—S1	−2.0 (4)
C2—C3—C4—N1	179.7 (3)	C10—C11—C12—S1	176.4 (2)
C2—C3—C4—C5	0.5 (5)	C11—C12—C13—C14	−177.5 (3)
N1—C4—C5—C6	−179.3 (3)	S1—C12—C13—C14	0.3 (4)
C3—C4—C5—C6	0.0 (5)	C12—C13—C14—C15	−1.0 (4)
C4—C5—C6—C1	−0.9 (5)	C13—C14—C15—S1	1.2 (4)
C2—C1—C6—C5	1.3 (5)	C3—C4—N1—C7	179.1 (3)
C9—C1—C6—C5	−179.3 (3)	C5—C4—N1—C7	−1.7 (5)
C6—C1—C9—C10	−2.8 (5)	C3—C4—N1—C8	11.2 (5)
C2—C1—C9—C10	176.5 (3)	C5—C4—N1—C8	−169.6 (3)
C1—C9—C10—C11	179.2 (3)	C14—C15—S1—C12	−0.9 (3)
C9—C10—C11—O1	−4.9 (5)	C13—C12—S1—C15	0.3 (3)
C9—C10—C11—C12	176.7 (3)	C11—C12—S1—C15	178.4 (3)

### Hydrogen-bond geometry (Å, º)

*Cg* is the centroid of the S1/C12–C15 thiophene ring.

**Table:**

*D*—H···*A*	*D*—H	H···*A*	*D*···*A*	*D*—H···*A*
C13—H13···O1^i^	0.95	2.65	3.451 (4)	142
C14—H14···S1^i^	0.95	3.00	3.779 (3)	141
C15—H15···O1^ii^	0.95	2.57	3.291 (4)	133
C8—H8···*Cg*^iii^	0.98	2.64	3.457 (4)	141

Symmetry codes: (i) *x*+1, *y*, *z*; (ii) −*x*−1/2, *y*−1/2, −*z*+3/2; (iii) −*x*, −*y*, −*z*+1.
