# Supplementary material for: Crystal structure of (2E)-3-[4-(di­methyl­amino)­phen­yl]-1-(thio­phen-2-yl)prop-2-en-1-one
Source: Acta Crystallogr E Crystallogr Commun. 2017 Mar 7;73(Pt 4):476–80. doi: 10.1107/S2056989017003437 (PMC5382602; doi:10.1107/S2056989017003437)
Supplement: Supplementary file 3 [file e-73-00476-sup3.pdf]

SUPPORTING INFORMATION FOR THE *IN SILICO* EVALUATION OF THE CRYSTAL STRUCTURE OF (2*E*)-3-[4-(DIMETHYLAMINO)PHENYL]-1-(2-THIENYL)-2-PROPEN-1-ONE AND THE NEURAMINIDASE ENZYME

by the co-author

Renan Lira de Farias

Instituto de Química

Universidade Estadual Paulista

Brazil

PART I

The protein sequence of H1N1 neuraminidase (ID: Q59311) was obtained from NCBI (NCBI Resource Coordinators, 2017).

SWISS-MODEL Template Library (SMTL) was used for the *in silico* calculations (Biasini *et al.*, 2014 & Sahoo *et al.*, 2016).

Three models (templates) were employed to predict the 3-D structure of the neuraminidase protein: PDB 3TI4 (resolution = 1.6 Å), PDB 4B7R (resolution = 1.9 Å) and PDB 4B7M (resolution = 2.5 Å). The alignment of the templates primary sequences and the targets sequences amount to 99.48 %, 99.48 % and 99.57 %, respectively (Fig. 1-Supporting Information). The sequence similarity for the three structures amounts to 63 %. The minimal accepted homology for *in silico* evaluations is 30 % (D'Alfonso *et al.*, 2001 & Vitkup *et al.*, 2001).

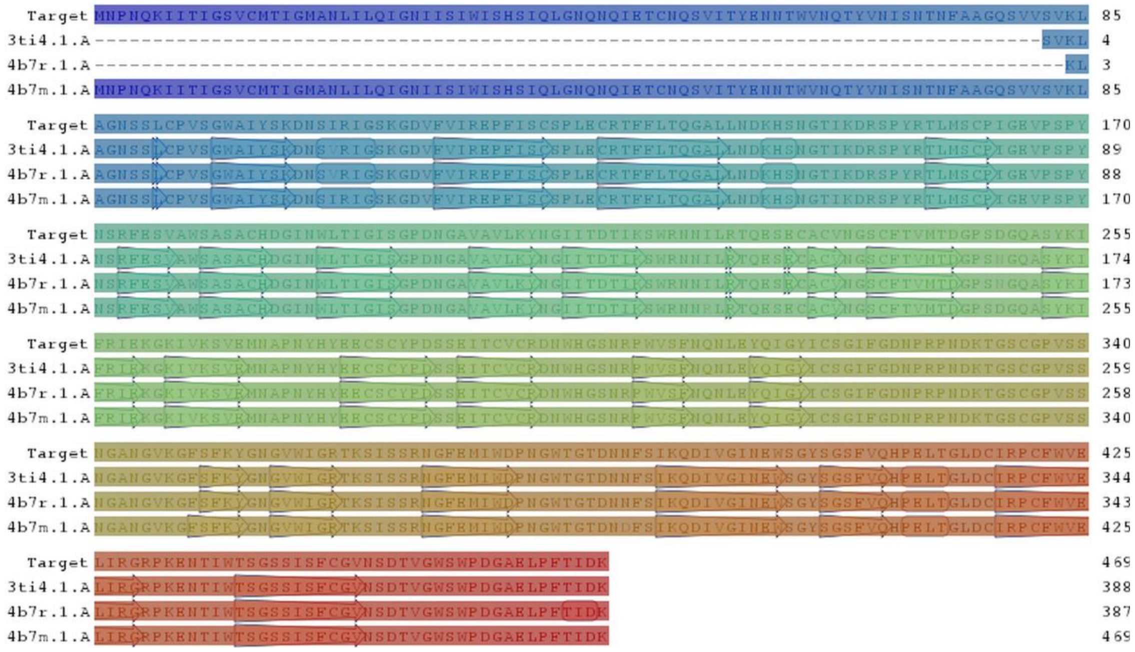

Figure 1-Supporting Information: alignment of sequence-target and sequence-templates for the neuraminidase PDB 3TI4, PDB 4B7R and PDB 4B7M structures (for details, see the references).

PART II

Ramachandran two-dimensional plot (Fig. 2-Supporting Information) (Anderson, 2003 & Lovell *et al.*, 2003).

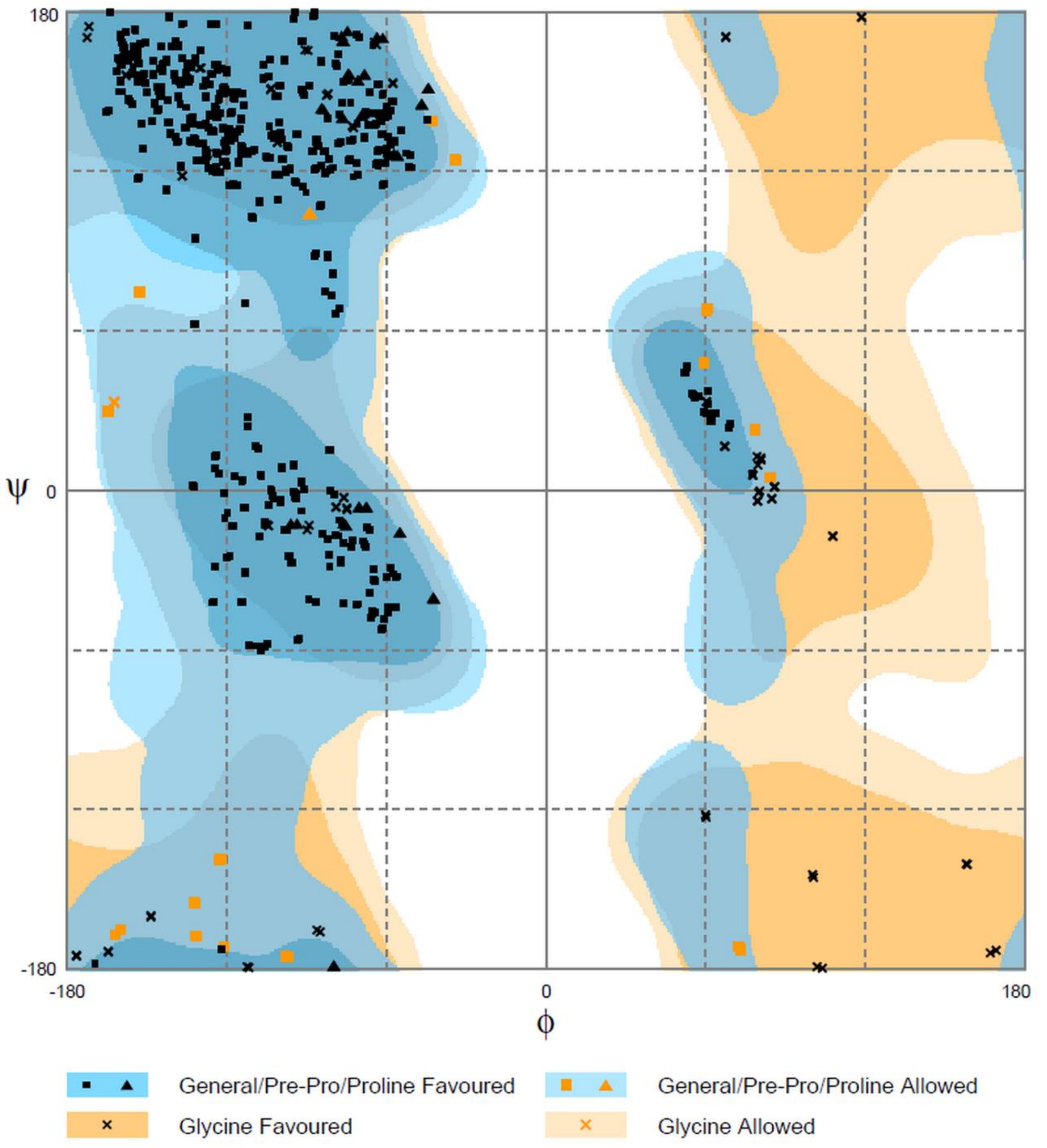

Figure 2-Supporting Information: stereochemical evaluation of the predicted neuraminidase model by the Ramachandran Plot (95.8 % of the residues are located in favoured regions, 4.2 % of the residues are located in allowed regions and there are no residues in outlier regions).

PART III

Z-score graph (Fig. 3-Supporting Information). The value for the neuraminidase model amounts to -5.09 (Wiederstein & Sippl, 2007).

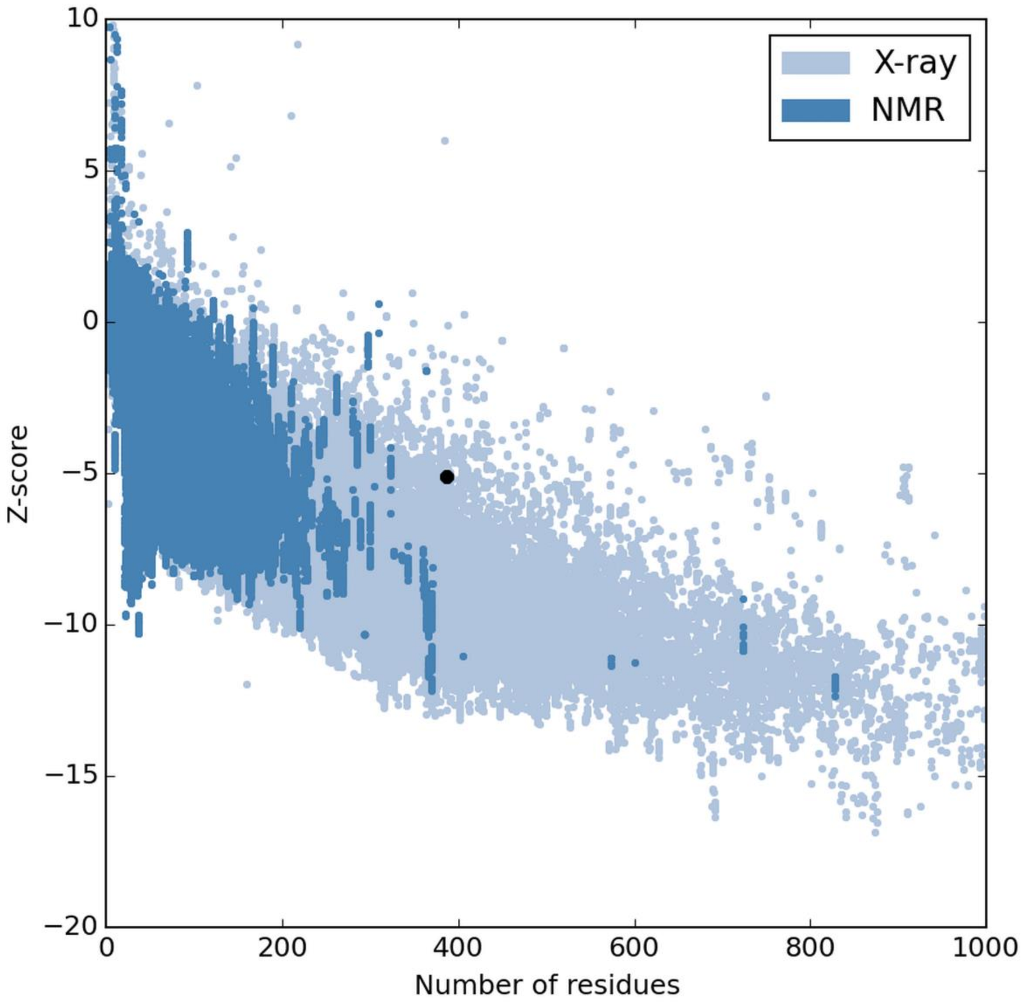

Figure 3-Supporting Information: Z-score graph for the predicted neuraminidase model (black dot).

PART IV

Neuraminidase 3-D structure based in the molecular modelling and classical validation (PARTS I, II, III and the respective references) (Fig. 4-Supporting Information).

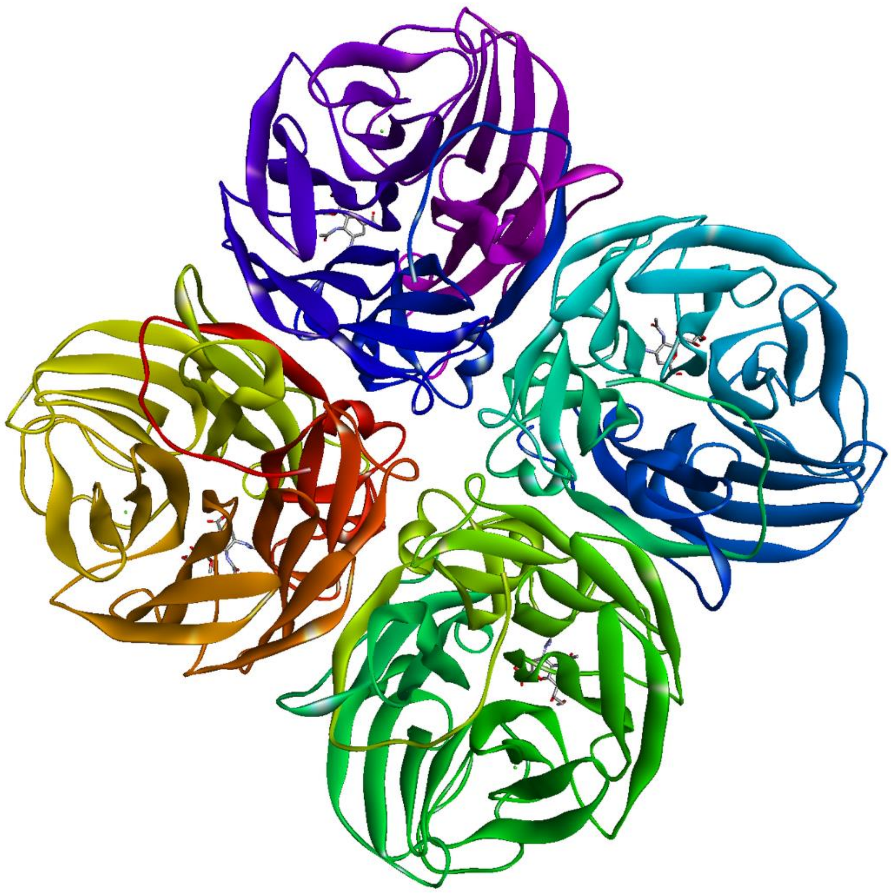

Figure 4-Supporting Information: graphical representation of the protein structure of the neuraminidase.

PART V

The employed docking methodology was validated by a re-docking of the co-crystallized inhibitor (Laninavir IC_{50} = 0.947μM) with the best X-ray resolution (PDB: 3TI4, resolution = 1.6 Å) of the neuraminidase protein. The best *in silico* conformation amounts to 86.43 *ChemPLP Fitness score* and the lowest RMSD amounts to 0.663 Å (Vavricka *et al.*, 2011) (Fig. 5-Supporting Information).

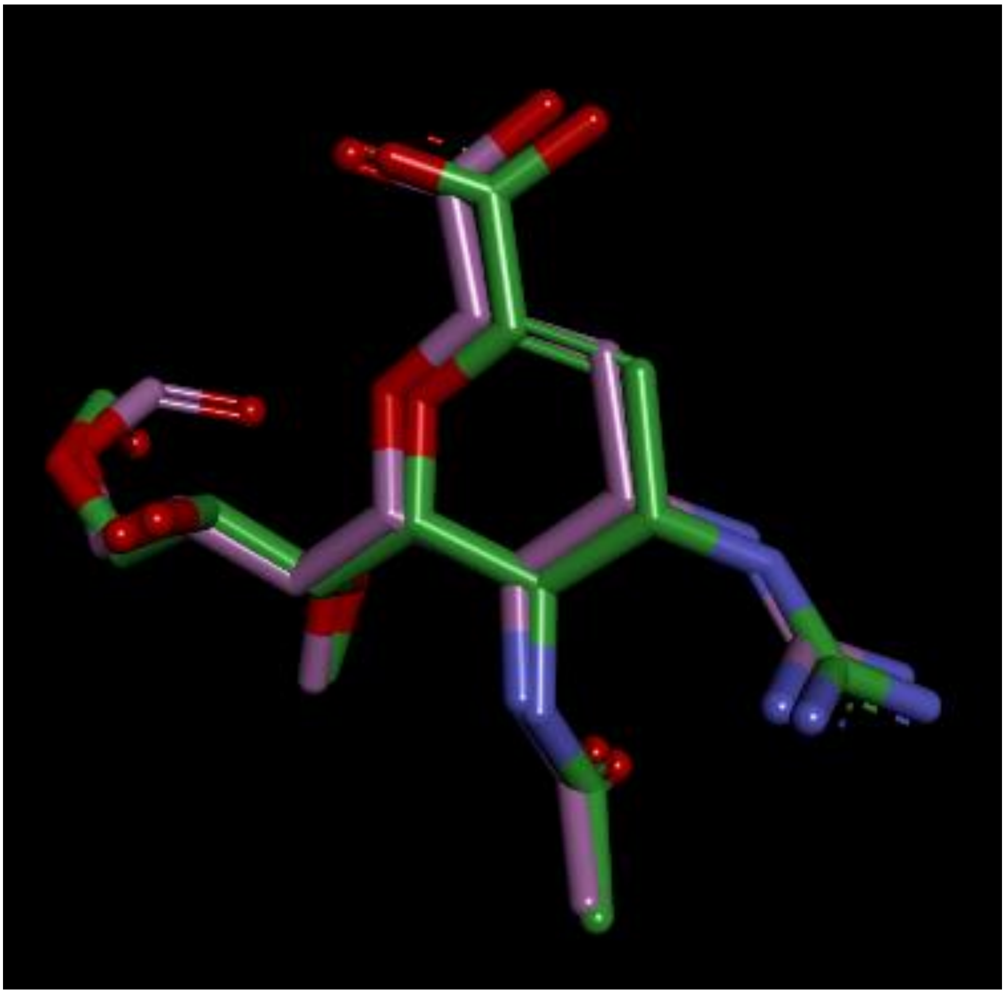

Figure 5-Supporting Information: re-docking of the Laninavir (neuraminidase inhibitor) in the active site of the related enzyme with PDB: 3T14. For the experimental conformation, the carbon atoms are drawn in green, while for the computational conformation, the carbon atoms are drawn in magenta. Oxygen and nitrogen atoms are drawn in red and blue for both of the conformations, respectively. The figure is simplified for clarity.

PART VI

References.

Anderson, A. C. (2003). *Chem. Biol.* **10**, 787-797.

Biasini, M., Bienert, S., Waterhouse, A., Arnold, K., Studer, G., Schmidt, T., Kiefer, F., Gallo Cassarino, T., Bertoni, M., Bordoli, L. & Schwede, T. (2014). *Nucleic Acids Res.* **42**, W252-258.

D'Alfonso, G., Tramontano, A. & Lahm, A. (2001). *J. Struct. Biol.* **134**, 246-256.

Lovell, S. C., Davis, I. W., Arendall, W. B., de Bakker, P. I., Word, J. M., Prisant, M. G., Richardson, J. S. & Richardson, D. C. (2003). *Proteins* **50**, 437-450.

NCBI Resource Coordinators (2017). *Nucleic Acids Res.* **45**, D12-D17.

Sahoo, M., Jena, L., Daf, S. & Kumar, S. (2016). *Genomics Inform.* **14**, 104-111.

Vavricka, C. J., Li, Q., Wu, Y., Qi, J., Wang, M., Liu, Y., Gao, F., Liu, J., Feng, E., He, J., Wang, J., Liu, H., Jiang, H. & Gao, G. F. (2011). *PLoS Pathog.* **7**, e1002249.

Vitkup, D., Melamud, E., Moult, J. & Sander, C. (2001). *Nat. Struct. Biol.* **8**, 559-566.

Wiederstein, M. & Sippl, M. J. (2007). *Nucleic Acids Res.* **35**, W407-410.
